# Semisynthetic aurones inhibit tubulin polymerization at the colchicine-binding site and repress PC-3 tumor xenografts in nude mice and myc-induced T-ALL in zebrafish

**DOI:** 10.1038/s41598-019-42917-0

**Published:** 2019-04-23

**Authors:** Yanqi Xie, Liliia M. Kril, Tianxin Yu, Wen Zhang, Mykhaylo S. Frasinyuk, Svitlana P. Bondarenko, Kostyantyn M. Kondratyuk, Elizabeth Hausman, Zachary M. Martin, Przemyslaw P. Wyrebek, Xifu Liu, Agripina Deaciuc, Linda P. Dwoskin, Jing Chen, Haining Zhu, Chang-Guo Zhan, Vitaliy M. Sviripa, Jessica Blackburn, David S. Watt, Chunming Liu

**Affiliations:** 1 Department of Molecular and Cellular Biochemistry, College of Medicine, University of Kentucky, Lexington, KY 40536-0509 USA; 2 Center for Pharmaceutical Research and Innovation, College of Pharmacy, University of Kentucky, Lexington, KY 40536-0596 USA; 3 Lucille Parker Markey Cancer Center, University of Kentucky, Lexington, KY 40536-0093 USA; 4 Institute of Bioorganic Chemistry and Petrochemistry, National Academy of Science of Ukraine, Kyiv, 02094 Ukraine; 5 National University of Food Technologies, Kyiv, 01601 Ukraine; 6 Center for Drug Innovation and Discovery, Hebei Normal University, Shijiazhuang, Hebei 050024 People’s Republic of China; 7 Department of Pharmaceutical Sciences, College of Pharmacy, University of Kentucky, Lexington, KY 40536-0596 USA; 8 Molecular Modeling and Pharmaceutical Center, College of Pharmacy, University of Kentucky, Lexington, KY 40536-0596 USA

**Keywords:** Drug development, Small molecules

## Abstract

Structure-activity relationships (SAR) in the aurone pharmacophore identified heterocyclic variants of the (*Z*)-2-benzylidene-6-hydroxybenzofuran-3(2*H*)-one scaffold that possessed low nanomolar *in vitro* potency in cell proliferation assays using various cancer cell lines, *in vivo* potency in prostate cancer PC-3 xenograft and zebrafish models, selectivity for the colchicine-binding site on tubulin, and absence of appreciable toxicity. Among the leading, biologically active analogs were (*Z*)-2-((2-((1-ethyl-5-methoxy-1*H*-indol-3-yl)methylene)-3-oxo-2,3-dihydrobenzofuran-6-yl)oxy)acetonitrile (**5a**) and (*Z*)-6-((2,6-dichlorobenzyl)oxy)-2-(pyridin-4-ylmethylene)benzofuran-3(2*H*)-one (**5b**) that inhibited *in vitro* PC-3 prostate cancer cell proliferation with IC_50_ values below 100 nM. A xenograft study in nude mice using 10 mg/kg of **5a** had no effect on mice weight, and aurone **5a** did not inhibit, as desired, the human ether-à-go-go-related (hERG) potassium channel. Cell cycle arrest data, comparisons of the inhibition of cancer cell proliferation by aurones and known antineoplastic agents, and *in vitro* inhibition of tubulin polymerization indicated that aurone **5a** disrupted tubulin dynamics. Based on molecular docking and confirmed by liquid chromatography-electrospray ionization-tandem mass spectrometry studies, aurone **5a** targets the colchicine-binding site on tubulin. In addition to solid tumors, aurones **5a** and **5b** strongly inhibited *in vitro* a panel of human leukemia cancer cell lines and the *in vivo* myc-induced T cell acute lymphoblastic leukemia (T-ALL) in a zebrafish model.

## Introduction

The aurones comprise a family of plant-derived flavonoids that arise out of a mixed polyketide-shikimate pathway, contribute to the yellow coloration of certain flowers^1^ and possess a range of biological properties^2–4^ affecting organisms ranging from protazoans to mammals. The antineoplastic activity^5^ of several naturally occurring aurones led to studies of natural and semisynthetic aurones as inhibitors of *in vitro* cancer cell proliferation^6–8^, typically at low micromolar concentrations. Additional studies identified a panoply of roles at a molecular level: drug efflux modulators^2,9–15^ of P-glycoprotein (P-gp) or ATP-binding cassette sub-family G member 2 (ABCG2), modifiers of adenosine-receptor interactions^16,17^, DNA sission-promoters^18^, teleomerase inhibitors^19^, sphingosine-kinase inhibitors^20^, phosphatidylinositol-3-kinases (PI_3_−α) inhibitors^21^, cyclin-dependent kinase inhibitors^22^, inducers of cytoprotective NAD(P)H:quinone oxidoreductase-1^23^ (NQO1), and scavengers of reactive-oxygen-species^24^ (ROS). Although these findings suggested that aurones would disrupt biological systems non-specifically, our studies of the aurone pharmacophore identified heterocyclic variants of the (*Z*)-2-benzylidene-6-hydroxybenzofuran-3(2*H*)-one scaffold that possessed the low nanomolar *in vitro* potency, encouraging *in vivo* potency in mouse xenograft and zebrafish models, selectivity for the colchicine-binding site in tubulin^25–31^, and the absence of appreciable toxicity.

**Figure 1:**
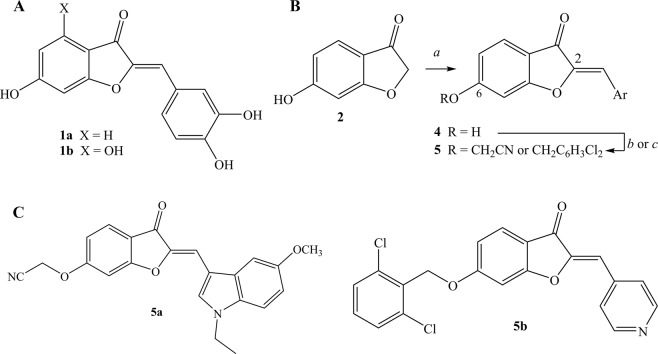
(**A**) Representative naturally occurring aurones, sulfuretin (**1a**) and aureusidin (**1b**). (**B**) Synthesis of aurones **4** and **5**. Legend: *a*, heterocyclic-substituted benzaldehydes or heteroaryl carboxaldehydes **3**, 50% aq. KOH, 1:1 EtOH:DMF, *b*, BrCH_2_CN, K_2_CO_3_, DMF; *c*, ClCH_2_C_6_H_3_-2,6-Cl_2_, K_2_CO_3_, DMF. (**C**) Biologically active aurones **5a** and **5b**.

Prior SAR studies of aurones as antineoplastic agents replaced the C-2 benzylidene subunit found in naturally occurring aurones, such as sulfuretin (**1a**) and aureusidin (**1b**) (Fig. 1A), with a C-2 heteroarylmethylene group. Aurones with 2-(coumarin-4-yl)methylene groups^32^ or 2-(furan-2-yl)methylene groups^33^ displayed *in vitro* activity against human leukemia K562 cells; aurones with 2-(piperazin-1-yl)methylene groups possessed IC_50_ values in the low micromolar range against various solid tumor cell lines^34^; and benzofuran-3(2*H*)-ones with 2-(indol-3-yl)methylene groups inhibited cell proliferation in breast cancer MCF-7 and MDA-MB-231 cell lines^35^. The relative potencies among these heterocyclic- and heteroarylmethylene-substituted aurones, the *in vivo* activity of these aurones, and the specific biological target or targets in these cases was unclear.

We determined that semisynthetic aurones with either 3-indolylmethylene or 4-pyridylmethylene groups at C-2 in place of the naturally occurring C-2 benzylidene group and with selected alkoxy groups at C-6 possessed *in vitro* potencies in the mid- to low nanomolar range using *in vitro* PC-3 cancer cell proliferation assays. The most potent of these aurones in these *in vitro* assays also displayed good activity in an *in vivo* PC-3 xenograft study. Although our studies focused on developing agents for the treatment of prostate cancers, the prior report that aurones with 2-(coumarin-4-yl)methylene groups^32^ or 2-(furan-2-yl)methylene groups^33^ displayed *in vitro* activity against human leukemia K562 cells prompted a study of myc-induced T-cell acute lymphoblastic leukemia (T-ALL) in a zebrafish model where these aurones also exhibited minimal toxicity. In summary, the aurones reported in this paper showed activity in two different animal models, displayed no apparent toxicity in two different species, and, like the literature reports cited above, showed activity against not only against prostate cancer PC-3 cells but also against leukemia cells. Finally, using a competition assay with mass spectrometry as an analytical tool, we established that these aurones functioned at a molecular level as tubulin polymerization inhibitors by binding to the colchicine-binding site.

## Results

### Synthesis of semisynthetic aurones

The condensation of 6-hydroxybenzofuran-3(2*H*)-one (**2**) with a spectrum of heteroaryl carboxaldehydes **3** under basic conditions led to aurones **4** (Fig. 1B). A mixture of 50% aqueous potassium hydroxide (2 eq) in 1:1 ethanol-*N*,*N*-dimethylformamide (DMF) was preferred over other conditions^23,36–38^ reported for similar condensations. The assignment of (*Z*)-stereochemistry in **4** was in accord with prior acid- or base-catalyzed condensations of benzofuran-3(2*H*)-ones with aromatic aldehydes^39,40^. The subsequent alkylation of the C-6 hydroxyl group in aurones **4** using various alkyl bromides and anhydrous potassium carbonate in DMF led to the 6-alkoxyaurones **5** (Fig. 1B).

### Structure-activity relationships (SAR)

A reiterative process of synthesis and screening using *in vitro* prostate cancer PC-3 cell proliferation assays identified an intersection of modifications at the C-2 and C-6 positions in semisynthetic aurones that were the most promising for further study (Table 1). Initial screening identified heteroarylmethylene-substituted aurones **4a**–**4d** with 1-isoquinolylmethylene, 2-quinolylmethylene, 8-methoxy-2-quinolylmethylene, and 5-methoxy-*N*-ethyl-3-indolylmethylene groups at C-2 and hydroxyl groups at C-6 as the most potent analogs at 10 μM concentrations but with only minimal activity at 1 μM concentrations (Table 1). Modifications at the C-4 and C-7 positions in the benzofuran ring in aurones **4** proved unrewarding in terms of increased potency (data not shown). Efforts to identify benzylidene-substituted aurones **4** with saturated, heterocyclic groups attached to the phenyl ring were equally unrewarding with the exception of (2*Z*)-6-hydroxy-2-(4-pyrrolidin-1-ylbenzylidene)-1-benzofuran-3(2*H*)-one (**4e**) (Table 1).

**Table 1:** Abbreviated SAR study involving modifications aurone at the C-2 and C-6 positions using prostate cancer PC-3 cell proliferation assays.

Aurone	C-6	C-2 Aryl or Heteroaryl Group	% Inhibition of PC-3 Cells		
			10 μM	1 µM	300 nM
**4a**	OH	1-isoquinolyl	88 ± 5.5		
**4b**	OH	2-quinolyl	99 ± 0.2	26 ± 8.6	
**4c**	OH	8-methoxy-2-quinolyl	97 ± 0.8	40 ± 8.4	
**4d**	OH	*N*-ethyl-5-methoxy-3indolyl	69 ± 16	13 ± 9.5	
**4e**	OH	4-(pyrrolidin-1-yl)phenyl	95 ± 4.7	2.1 ± 7.1	
**5a**	OCH_2_CN	*N*-ethyl-5-methoxy-3-indolyl		95 ± 2.5	93 ± 2.8
**5b**	OCH_2_C_6_H_3_-2,6-Cl_2_	4-pyridyl		92 ± 0.4	95 ± 1.1

**Figure 2:**
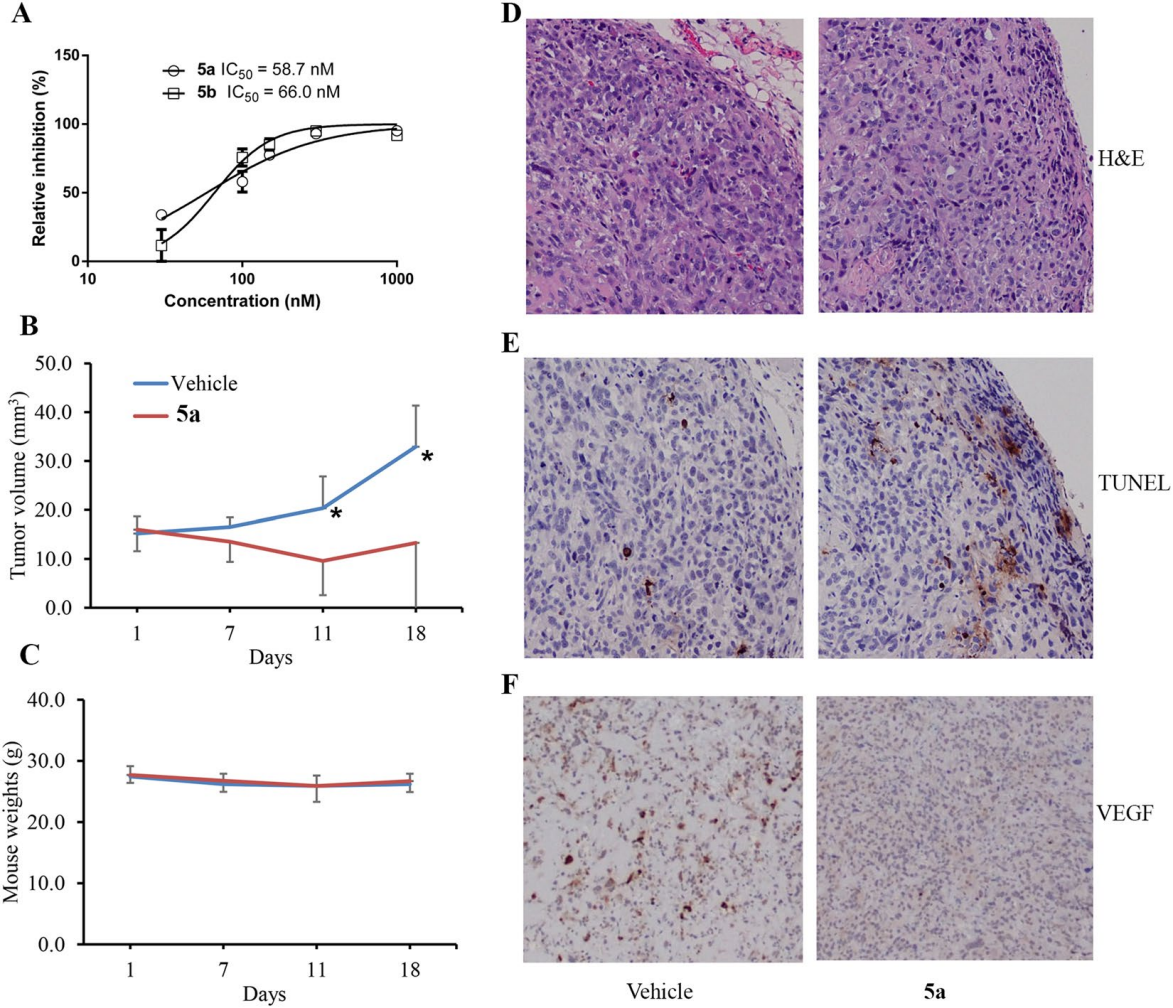
(**A**) Dose responses of aurones **5a** and **5b** in PC-3 cell proliferation inhibition assay. (**B**) Effect of aurone **5a** on PC-3 tumor xenografts in nude mice (n = 5) at 10 mg/kg/day. (**C**) Effect on aurone **5a** on body weights of the treated mice: *P < 0.05, t-test. (**D**) H&E analysis of tumor sections. (**E**). Apoptosis analysis by TUNEL assay. (**F**). IHC analysis of angiogenesis marker, VEGF-A.

Additional efforts to improve potency in aurones **4a**–**4e** led to the alkylation of the C-6 hydroxyl group with a range of alkylating agents to obtain 6-alkoxyaurones **5** (Fig. 1B). An SAR study involving dual modifications of the C-6 alkoxy group and the C-2 heteroarylmethyelene group identified two aurones with 90%^+^ inhibition of *in vitro* prostate cancer PC-3 cell proliferation at 300 nM concentration: (*Z*)-2-((2-((1-ethyl-5-methoxy-1*H*-indol-3-yl)methylene)-3-oxo-2,3-dihydrobenzofuran-6-yl)oxy)acetonitrile (**5a**) and (*Z*)-6-((2,6-dichlorobenzyl)oxy)-2-(pyridin-4-ylmethylene)benzofuran-3(2*H*)-one (**5b**) (Fig. 1C). In a dose-response study, aurone **5a** and **5b** displayed IC_50_ values of 58.7 ± 1.1 nM and 66 ± 1.1 nM (Fig. 2A), respectively. Aurone **5a** displayed an IC_50_ value of 1.3 ± 0.2 μM using *normal* human embryo lung HEL299 cells that indicated that aurone **5a** was selectively more toxic to a cancer cell line than a normal cell line.

The pairing of the cyanomethoxy group at C-6 with the (*N*-ethyl-5-methoxy-1*H*-indol-3-yl)methylene at C-2 in aurone **5a** and the pairing of the 2,6-dichlorobenzyloxy group at C-6 with the (pyridin-4-yl)methylene at C-2 in aurone **5b** (Fig. 1C) were essential to potency. Alternate pairings, modification in the halogenation type and pattern in the 2,6-dichlorobenzyloxy group, changes in the *N*-ethyl-5-methoxy-1*H*-indol-3-yl group (*e.g*., replacement of the *N*-ethyl with an *N*-methyl group; replacement of the 5-methoxy with a 5-hydroxy group), and modifications at still other positions in the benzofuran (*e.g*., methyl groups at C-7) led to diminished activity in the prostate cancer PC-3 cell proliferation assay relative to aurones **5a** and **5b**. Finally, we performed additional cell proliferation inhibition studies using other cancer cell lines, and aurones **5a** and **5b** showed potent low nanomolar activities against these cell lines (Table 2).

**Table 2:** IC_50_ values of aurones 5a and 5b in cancer cell line proliferation inhibition assays.

Cell lines	IC_50_ (nM)	
	Aurone 5a	Aurone 5b
PC-3	58.7 ± 1.1	66.0 ± 1.1
LS174T	155.2 ± 1.1	158.3 ± 1.0
A549	173.6 ± 1.0	113.0 ± 1.0
MCF-7	244.3 ± 1.2	185.6 ± 1.1
NCI/ADR-res	85.9 ± 1.0	190.3 ± 1.1
OVCAR-8	181.9 ± 1.0	257.7 ± 1.1

### Prostate cancer PC-3 xenograft study in mice using aurone 5a

We evaluated the *in vivo* tumor inhibitory effect of aurone **5a** using prostate cancer PC-3 xenografts in immune-defective nude mice. PC-3 cells were subcutaneously injected into both flanks of nude mice. Two weeks after the inoculation, the mice were randomized to two groups (n = 5), treated with aurone **5a** or control vehicle by intraperitoneal administration for 18 days and then sacrificed. Compared to vehicle, the administration of **5a** at 10 mg/kg/day showed significant, tumor-growth suppression (Fig. 2B). Importantly, aurone **5a** achieved tumor regression with no apparent gross toxicity as reflected by minimal changes in mice weights (Fig. 2C). To understand the mechanisms of aurone **5a**-induced tumor repression, we performed another PC-3 xenograft study by treating the tumors with vehicle and aurone **5a** for one week. Tumor sections were analyzed by H&E (Fig. 2D) and immunohistochemistry (IHC) stainings (Fig. 2E,F). We observed increased apoptosis (Fig. 2E) and decreased angiogenesis marker, VEGF (Fig. 2F), in aurone **5a**-treated tumors.

### Effect of aurone 5a on tubulin polymerization

**Figure 3:**
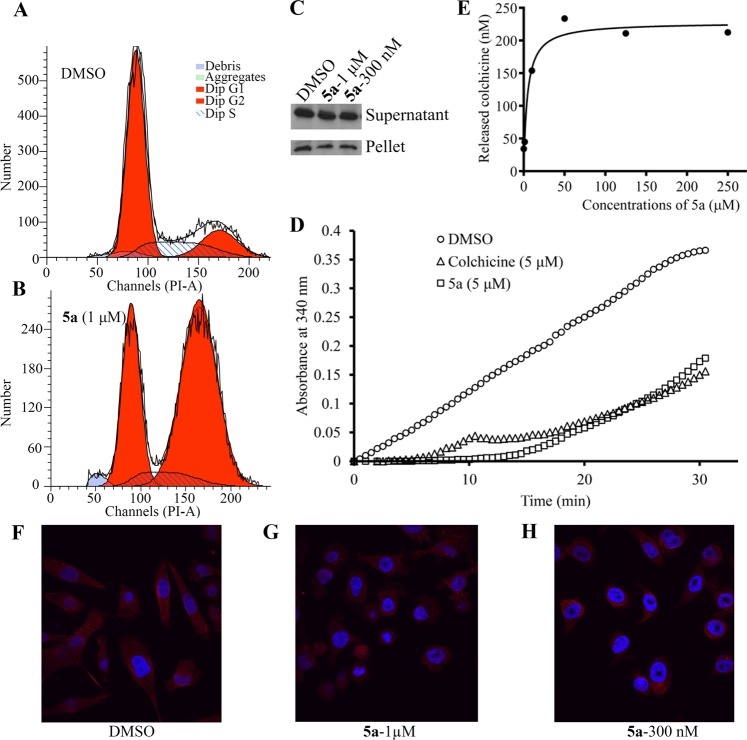
(**A**,**B**) Aurone **5a** induced cell cycle arrest. (**C**) Aurone **5a** decreased tubulin polymerization. (**D**) Aurone **5a** (5 µM) and colchicine (5 µM) inhibited tubulin polymerization *in vitro* in a similar fashion. (**E**) Competitive tubulin binding assay with colchicine in the presence of increasing concentrations of aurones **5a**. (**F**–**H**) Aurone **5a** treatment (6 h) inhibited microtubule structures and caused cell morphology change in PC-3 cells as shown in panels F, DMSO; G, **5a** (1 µM); H, **5a** (300 nM). Red immunofluorescence: α-tubulin; blue: DAPI.

An analysis of the screening data (Table 3) from the NCI-60 human tumor cell lines available through the developmental therapeutics program of NCI showed excellent response to aurone **5a** with IC_50_ values in the range of 200–500 nM. These values were consistent with the IC_50_ values determined by Vi-CELL XR 2.03 (Fig. 2A and Table 2). An analysis of the NCI-60 data from aurone **5a** using the COMPARE algorithm^41^ matched the response of cell lines to aurone **5a** with the response of other tubulin-polymerization inhibitors. An analysis of the effects of aurone **5a** on cell cycle progression using PC-3 cells indicated significant cell cycle arrest at G2/M phases (Fig. 3A,B), again consistent with the inhibition of tubulin microtubule assembly. We then investigated the level of tubulin polymerization in PC-3 cells treated by aurone **5a** at indicated concentrations. After cell treatment and lysis, we separated the cell lysates by centrifugation into supernatants and pellets, that were individually subjected to western blotting using antibodies against β-tubulin. After treatment with aurone **5a** for 6 hours, the amount of tubulin in pellets was significantly less than that in cell lysates from dimethyl sulfoxide (DMSO)-treatment alone, even at a concentration as low as 300 nM (Fig. 3C). We also performed an *in vitro* tubulin polymerization assay in the presence and absence of aurone **5a**. In the presence of glycerol and guanosine triphosphate, either aurone **5a** at 5 µM or colchicine at 5 µM decreased the formation of microtubules in a similar fashion whereas a DMSO-treated control group showed, as expected, substantial tubulin polymerization (Fig. 3D).

**Table 3:** IC_50_ values of aurone 5a in NCI-60 cell line proliferation inhibition assays (Data were produced by the Nation Cancer Institute (Maryland, USA).

Panel/Cell Line	GI_50_ (nM)	Panel/Cell Line	GI_50_ (nM)
Leukemia		Melanoma	
CCRG-CEM	289	LOX IMVI	696
HL-60(TB)	236	MALME-3M	>100 µM
K-562	212	M14	319
MOLT-4	523	MDA-MB-435	174
RPMI-8226	352	SK-MEL-2	836
SR	275	SK-MEL-28	10.2 µM
**Non-Small Cell Lung Cancer**	**GI**_**50**_ **(nM)**	SK-MEL-5	405
		UACC-257	67.1 µM
A549(ATCC)	5.1 µM	UACC-62	499
EKVX	2.73 µM	**Ovarian Cancer**	**GI**_**50**_ **(nM)**
HOP-62	542		
HOP-92	NA	IGROV1	774
NCI-H226	57.4 µM	OVCAR-3	377
NCI-H23	812	OVCAR-4	19 µM
NCI-H322M	1.43 µM	OVCAR-5	2.52 µM
NCI-H460	337	OVCAR-8	483
NCI-H522	3.13 µM	NCI/ADR-RES	406
**Colon Cancer**	**GI**_**50**_ **(nM)**	SK-OV-3	669
		**Renal Cancer**	**GI**_**50**_ **(nM)**
COLO 205	446		
HCC-2998	3.44 µM	786-0	470
HCT-116	386	A498	10.3 µM
HCT-15	399	ACHN	794
HT29	356	RXF 393	182
KM12	546	SN 12C	763
SW-620	345	TK-10	56.9 µM
**CNS Cancer**	**GI**_**50**_ **(nM)**	UO-31	864
		**Breast Cancer**	**GI**_**50**_ **(nM)**
SF-268	848		
SF-295	307	MCF7	311
SF-539	269	MDA-MB-231	2.66 µM
SNB-19	468	HS 578T	360
SNB-75	5.65 µM	BT-549	571
U251	453	T-47D	NA
**Prostate Cancer**	**GI**_**50**_ **(nM)**	MDA-MB-468	2.16 µM
PC-3	367		
DU-145	643

### Competition study of colchicine and aurone 5a for the colchicine-binding site on tubulin

A competitive, tubulin-binding assay^42^ confirmed that aurone **5a** bound to the colchicine-binding site. Aurone **5a** was added at various concentrations to a solution of α/β-tubulins (1.3 mg/mL) and colchicine (1.25 μM). Unbound colchicine was separated from either tubulin-colchicine or tubulin-aurone **5a** complex by Amicon Ultra-0.5 mL Centrifugal Filters (30 kDa Cut-off). The level of unbound colchicine was measured by liquid chromatography-electrospray ionization-tandem mass spectrometry (LC-MS/MS). Aurone **5a** released colchicine from tubulin in a dose-dependent manner (Fig. 3E) that indicated that aurone **5a** bound to the colchicine-binding site on tubulin.

### Effects of aurone 5a on microtubule networks

We analyzed microtubule networks in PC-3 cells by immunofluorescence staining using an anti-α-tubulin Ab (Fig. 3F–H). The control, DMSO-treated cells retained their normal microtubule network and an overall, shuttle-like morphology (Fig. 3F) whereas aurone **5a**-treated cells demonstrated significant microtubule depolymerization and adopted a round morphology (Fig. 3G,H).

### Molecular docking analysis

**Figure 4:**
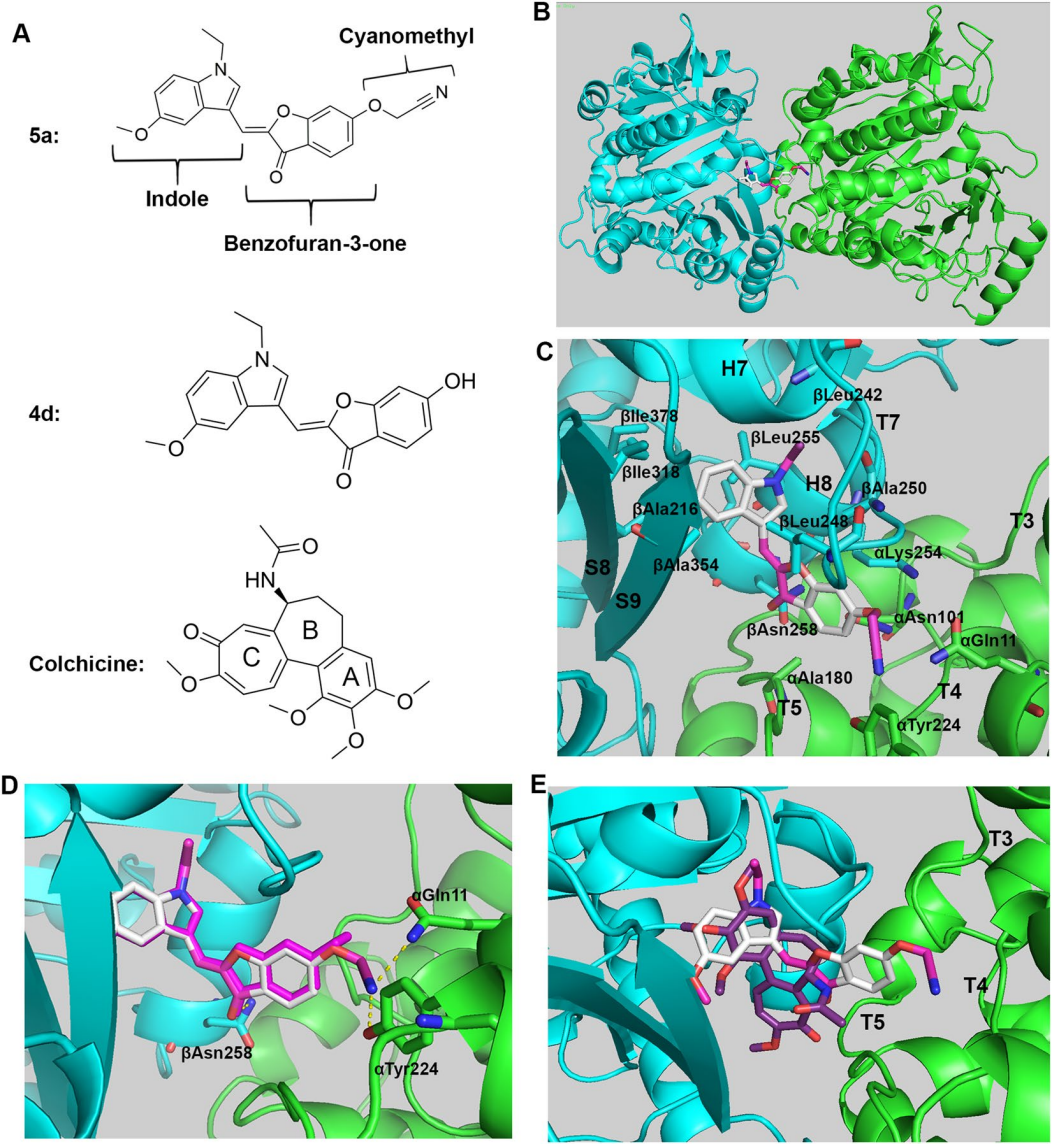
**(A**) Structures of aurone **5a**, a less active aurone **4d**, and colchicine. **(B**) Aurone **5a** bound to the colchicine-binding site (CBS) in the interface of αβ-tubulin dimers (cyan for β, green for α). **(C**) Close-up view of the interaction environment of **5a** (gray sticks) and tubulin (carton). **(D**) Superimposition of **5a** (gray sticks) and **4d** (magenta sticks) in the colchicine-binding site. Hydrogen bonding is represented by yellow, dashed lines. **(E**) Superimposition of **5a** (gray sticks) and colchicine (purple sticks) in the colchicine-binding site.

We performed molecular docking using AutoDock Vina^43^ to explore the possible binding of aurone **5a** to the colchicine-binding site (CBS) on αβ-tubulin heterodimers because this site was well known to host a plethora of chemically unrelated compounds^44^. A less active aurone **4d** (Fig. 4A) than aurone **5a** and colchicine were also docked into the CBS for comparison. We observed that aurone **5a**, **4d** and colchicine occupied the CBS at the interface of the α-tubulin and α-tubulin heterodimer (Fig. 4B). A hydrophobic pocket formed by Ala, Ile and Leu residues from β-tubulin accommodated the hydrophobic indole moiety of aurone **5a** (Fig. 4C**)**. The benzofuran-3(2*H*)-one and cyanomethoxy groups in aurone **5a** participated in hydrophobic contacts with the loop T7 and helix H8 of β-tubulin and with the loops T3, T4 and T5 from α-tubulin (Fig. 4C)^45^. In addition, hydrogen-bonding interactions between the carbonyl oxygen of the benzofuran-3(2*H*)-one and βAsn258 and hydrogen-bonding interactions between the nitrogen of the cyanomethoxy group and αTyr224 and αGln11 provided additional binding stabilization (Fig. 4D).

The indole moiety and a portion of the benzofuran-3(2*H*)-one in aurone **5a** superimposed well with the colchicine A and B rings (Fig. 4E); however, aurone **5a** did not occupy the hydrophobic pocket within β-tubulin in which the colchicine C ring resided. Instead, aurone **5a** formed contacts with loops T3, T4, and T5 of α-tubulin using the benzofuran-3(2*H*)-one and cyanomethoxy groups. Additionally, a comparison of the binding poses of **5a** and **4d** revealed why **5a** possessed better potency than **4d**. Aurone **4d** had major interactions with β-tubulin but lacked the bifurcated, hydrogen-bonding between the nitrogen of the cyanomethoxy group and αTyr224 and αGln11 of α-tubulin. This deficiency weakened the binding affinity of aurone **4d** relative to the potent aurone **5a** (Fig. 4D).

### Leukemia cell study in zebrafish using aurone 5a

**Figure 5:**
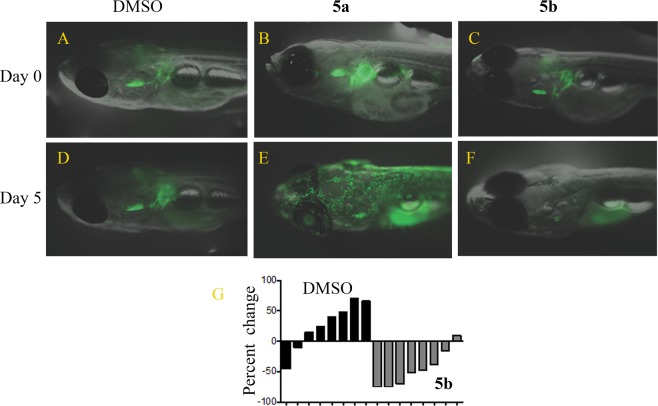
Aurones **5a** and **5b** inhibited myc-induce T-ALL in a zebrafish model. (**A**,**D**) Treatment of GFP-labeled thymic lymphoma cells with DMSO alone at day 0 and day 5, respectively. (**B**,**E**) Treatment of GFP-labeled thymic lymphoma cells with aurone **5a** in DMSO at day 0 and day 5, respectively. (**C**,**F**) Treatment of GFP-labeled thymic lymphoma cells with aurone **5b** at day 0 and day 5, respectively. (**G**) Percent change in fluorescence (*i.e*., number of GFP-labeled thymic lymphoma cells) as a function of time from administration of DMSO alone to the administration of aurone **5b** in each zebrafish (n = 8).

In addition to the *in vivo* PC-3 xenograft study in mice, we sought to test these aurones in a second species. Two prior reports indicated that aurones with 2-(coumarin-4-yl)methylene groups or 2-(furan-2-yl)methylene groups displayed *in vitro* activity against a leukemia cell line. Consequently, we tested various leukemia cell lines and found that the IC_50_ values for aurone **5a** were in the mid-nanomolar range (Table 4). The IC_50_ values of two normal B-lymphoblast cells were much higher than the leukemia cell lines and suggested a preferential toxicity of aurone **5a** toward leukemia cells. Because these leukemia cell lines had various mutations, we tested the activity of aurone **5a** *in vivo* using a genetically well-defined, zebrafish myc-induced T-ALL leukemia model^46,47^ (Fig. 5). The zebrafish (*Danio rerio*) is a vertebrate system that develops tumors similar to those in humans and that provides a plaform that is easy to manipulate for *in vivo* assays even in large-scale screens. According to previous studies^46,47^, the zebrafish *Rag2* promotor controlling the *myc-GFP* transgene specifically targets gene expression to lymphoid cells. The *Rag2*: *myc-GFP* transgene was micro-injected into wild-type zebrafish embryos at the one-cell development stage, and a small fraction of injected embryos developed *c-myc* induced leukemia. We treated GFP-labeled leukemia cells in zebrafish with either DMSO (Fig. 5A at day 0 and **5D** at day 5); aurone **5a** in DMSO (Fig. 5B at day 0 and **5E** at day 5); or aurone **5b** in DMSO (Fig. 5C at day 0 and **5 F** at day 5). Since aurone **5a** had auto-fluorescence that interfered with visualizing the loss of the GFP-labeled leukemia cells (Fig. 5E), we selected aurone **5b** that lacked this auto-fluorescence and clearly displayed the loss of the GFP-labeled leukemia cells (Fig. 5F). Aurone **5a** and **5b** significantly blocked the progression of T-ALL in zebrafish (Fig. 5D *versus* **5F**, Fig. 5G).

**Table 4:** IC_50_ values of aurone 5a in leukemia cell line proliferation inhibition assays.

Cell Line	Cell Type	IC_50_ (nM)	95% Confidence Interval (nM)
CCRF-CEM	T-ALL	244	197–301
DND41	T-ALL	210	116–379
Jurkat	T-ALL	273	226–344
HBP-ALL	T-ALL	94	51–173
Loucy	T-ALL	334	285–391
Molt-4	T-ALL	241	114–402
Molt-16	T-ALL	234	218–250
RPMI8402	T-ALL	301	248–364
Nalm-16	B-ALL	272	248–291
REH	B-ALL	287	252–326
NCI-BL2009a	Normal B-Lymphoblast	1,253	429–3,658
HCC1007-BL	Normal B-Lymphoblast	1,379	372–2,490

### Interaction of aurone 5a with potassium channel derived from human ether-a-go-go related gene (*hERG*)

Inhibition of the hERG potassium channel derived from *hERG* often leads to drug failure in preclinical studies or even in clinical trials. We utilized a well-established [^3^H]-dofetilide binding assay^48^ to evaluate the interaction of aurones with hERG. [^3^H]-Dofetilide competition binding assays using HEK-293 cell membranes stably expressing the hERG channel (hERG-HEK) correlated well with results from voltage-clamp assays and provided useful predictive screening assays for QT prolongation^49^. Amitriptyline (final concentration, 1 mM) was used as the positive control and exhibited an IC_50_ value (10.7 ± 2.25 μM) in agreement with published values^50^. Concentrations of aurones **5a** and **5b** ranging from 10^−9^ to 10^−4^ M were assayed in duplicate for these experiments (n = 3 experiments/analog). As desired, aurones **5a** and **5b** displayed no hERG inhibition (IC_50_ values > 100 μM).

## Discussion

Two types of inhibitors target tubulin microtubule dynamics: stabilizing agents, such as paclitaxel, and destabilizing agents, such as the Vinca alkaloids and colchicine. These agents bind tubulin subunits at well-characterized, binding sites, some of which find broad application in cancer therapeutics, including prostate cancer. Until recently, few agents were known that targeted the colchicine-binding site, but various pharmacophores^25–31^ now appear to exhibit excellent potency and selective binding to the colchicine-tubulin site. The impetus for developing these agents derives in part from the continuing need for new tubulin-targeting drugs to meet the needs of patients experiencing resistance or developing mutations crippling the use of traditional taxol or Vinca-based therapies. The semisynthetic aurones reported here provide a new pharmacophore for the development of colchicine-targeting microtubule inhibitors for cancer treatment.

Prior reports that naturally occurring aurones, such as sulfuretin (**1a**) and aureusidin (**1b**) (Fig. 1A), and several semisynthetic aurones possessed *in vitro* antineoplastic activity encouraged our interest in exploring SAR relationships within the aurone pharmacophore. A straightforward condensation of 6-hydroxybenzofuran-3(2*H*)-one (**2**) with various aryl or heteroaryl carboxaldehydes **3** furnished aurones **4** in which the C-2 benzylidine groups were either substituted with or replaced by heterocycles (Fig. 1B). Using a PC-3 cell proliferation assay as a readout, we found that the aurones **4a-4e** bearing nitrogen-containing heterocycles at C-2 were marginally active in a 1–10 μM concentration range (Table 1). Alkylation of the C-6 hydroxyl group in concert with alterations in the C-2 heteroarylmethylene subunit led ultimately to two aurones **5a** and **5b** (Fig. 1C) with IC_50_ values of 58.7 ± 1.1 nM and 66 ± 1.1 nM, respectively (Fig. 2A). The pairing of the unusual cyanomethoxy group at C-6 with the (*N*-ethyl-5-methoxy-1*H*-indol-3-yl)methylene at C-2 in aurone **5a** and the pairing of the 2,6-dichlorobenzyloxy group at C-6 with the (pyridin-4-yl)methylene at C-2 in aurone **5b** were essential to achieve nanomolar potency.

In addition to these *in vitro* studies, we evaluated the *in vivo* tumor inhibitory effect of aurone **5a** using prostate cancer PC-3 xenografts in immune-defective nude mice. Compared to vehicle, the administration of aurone **5a** at 10 mg/kg/day showed significant, tumor-growth suppression (Fig. 2B). Importantly, aurone **5a** achieved tumor regression with no apparent gross toxicity as reflected by minimal changes in mice weights (Fig. 2C). IHC staining suggested that aurone **5a** treatment induced apoptosis and decreased angiogenesis in the xenografted tumors (Fig. 2E,F), which is consistent with the function other microtubule inhibitors^51^. In summary, SAR studies identified aurone **5a** that possessed good *in vitro* activity in cancer cell proliferation studies in the nanomolar range, good reduction in tumor volume in an *in vivo* prostate PC-3 xenograft study, and minimal gross toxicity based on minimal weight loss during the *in vivo* studies. Preliminary indications involving the minimal effects on normal cell proliferation, the minimal changes in mice weights during xenograft studies, the absence of hERG inhibition and absence of toxic effects on zebrafish in studies, as described below, suggested that aurone **5a** had an acceptable “toxicity window” that was sufficient to warrant further study.

Knowledge about the binding site between a ligand and its biological target is pivotal for structure-guided, rational design of compounds with improved properties including potency and solubility. Molecular docking studies showed that aurone **5a** binds to the colchicine-binding site between the α-tubulin and β-tubulin. The indole moiety and part of the benzofuran-3-one of aurone **5a** as well as the A and B rings of colchicine occupied a hydrophobic pocket in β-tubulin (Fig. 4C,E). However, aurone **5a** did not occupy another hydrophobic pocket in which the colchicine C ring normally resided. Instead, aurone **5a** interacted more with α-tubulin than β-tubulin and participated in bifurcated hydrogen-bonding between the nitrogen of the cyanomethoxy group and αTyr224 and αGln11 of α-tubulin (Fig. 4D). The relatively inactive aurone **4d** failed to form this same interaction because it lacked a cyanomethyl group.

To confirm that aurone **5a** bound to the colchicine-binding site, we performed a tubulin polymerization assay and a competitive tubulin-binding assay^42^. Aurone **5a** inhibited tubulin polymerization *in vitro* (Fig. 3D). In addition, aurone **5a** bound to the CBS, resulting in an increased amount of unbound colchicine (Fig. 3E). These data were consistent with molecular docking results, and echoed the fact that the CBS would accommodate chemically diverse compounds. Mechanistically, previous crystallography studies show that free tubulin dimers are in a “straight” state and polymerized tubulin dimers in microtubules are in a “curved” conformation^45,52–54^. During tubulin polymerization, tubulin dimers structurally transitioned from a straight state to a curved state, during which the T7 loop of β-tubulin flipped inwards into the CBS. As a mechanism of action, colchicine bound to the CBS, prevented the T7 loop flipping towards the CBS, and thus inhibited tubulin polymerization^53,55^. Importantly, our leading compounds showed strong interaction with T7 loop (Fig. 4C,E) and reflected a similar mechanism of action seen with colchicine. As a result, aurone **5a** strongly inhibited cell cycle progression at G2/M phases (Fig. 3A,B) and disrupted microtubule networks in PC-3 cells (Fig. 3F–H).

By testing the efficacy of aurone **5a** in the NCI-60 and other cell lines, we found that **5a** demonstrated broad-spectrum, anticancer activity (Fig. 2A, Tables 2–4). The NCI/ADR-RES cell line that was normally resistant to adriamycin and many other cancer chemotherapeutics due to the expression of P-glycoprotein exhibited inhibition by aurone **5a**, and hence, aurone **5a** was not a likely substrate of P-glycoprotein. As previously noted, aurone **5a** showed no general toxicity in nude mice at doses that significantly inhibited PC-3 tumor xenografts (Fig. 2C). We also tested aurones **5a** and **5b** in zebrafish models where we again observed no gross toxicity on zebrafish but observed significant inhibition of myc-induced T-ALL *in vivo* (Fig. 5). The zebrafish myc-induced T-ALL model could be an important *in vivo* tool to screen and characterize future aurone analogs.

In summary, we identified two potent, semisynthetic aurones **5a** and **5b** that function as tubulin inhibitors with IC_50_ values of 58.7 ± 1.1 nM and 66 ± 1.1 nM, respectively (Fig. 2A). Importantly, aurone **5a** displayed activity in an *in vivo* PC-3 prostate cancer xenograft model in nude mice at 10 mg/kg without affecting mice weight (Fig. 2B,C). Aurones **5a** and **5b** showed potent *in vivo* activity in a genetically well-defined, zebrafish myc-induced T-ALL leukemia model^46,47^ (Fig. 5). Aurone **5a** also displayed no appreciable affinity for human hERG potassium channel and was not a substrate of P-glycoprotein. An analysis of screening data from the NCI-60 human tumor cell lines using the COMPARE algorithm^41^ matched the response to aurone **5a** with other tubulin-polymerization inhibitors. We used combination of experimental studies to examine this prediction: a competition study of colchicine and aurone **5a** for the colchicine-binding site on tubulin (Fig. 3E), a study of the comparative inhibition of tubulin polymerization with aurone **5a** and colchicine (Fig. 3D), and detailed computational modeling of the binding of these agents to tubulin (Fig. 4). Liquid chromatography-electrospray ionization-tandem mass spectrometry studies further confirmed that aurone **5a** targeted the colchicine-binding site on tubulin. Continued studies will define the pharmacokinetic and pharmacodynamics properties of aurones in this family.

### Chemistry Materials and Methods

Chemicals were purchased from Sigma-Aldrich (St. Louis, MO) or Fisher Scientific (Pittsburgh, PA) unless otherwise noted or were synthesized according to literature procedures. Solvents were used from commercial vendors without further purification unless otherwise noted. Nuclear magnetic resonance spectra were determined on Varian instruments (^1^H, 400 or 500 MHz; ^13^C, 100 or 126 Mz). Low-resolution mass spectra were obtained using an Agilent 1100 (atmospheric pressure, chemical ionization) instrument. High resolution mass data were obtained by direct infusion electrospray ionization mass spectrometry (-MS) using a LTQ-Orbitrap mass spectrometer coupled with a Heated Electrospray Ionization (HESI-II) Probe (Thermo Fisher Scientific, Waltham, MA) and an FT analyzer at a resolution of 100,000. The reported *m/z* mass was a mean of 20 scans. Melting points were determined in open capillarity tubes with a Buchi B-535 apparatus and are uncorrected. Compounds were purified by chromotography on preparative layer Merck silica gel F254 unless otherwise noted.

### General procedure for the synthesis of aurones 3a-3f and 4a-4o

To a suspension of 10 mmol of 6-hydroxybenzofuran-3(2*H*)-one (**2)** (Ark Pharm, Arlington Heights, IL USA) in 20 mL of a 1:1 mixture of DMF and absolute ethanol was added 2.3 mL of 50% aqueous potassium hydroxide. To this clear solution, obtained after stirring for *ca*. 30 min, was added 10 mmol of the appropriate carboxaldehyde. The mixture was stirred for 6–8 h at 25 °C. The mixture was diluted with 100 mL of hot water, acidified with glacial acetic acid pH 5. The resulting precipitate was collected by filtration, washed with water, dried and re-crystallized from DMF-methanol.

### (2*Z*)-6-Hydroxy-2-(isoquinolin-1-ylmethylene)-1-benzofuran-3(2*H*)-one (4a)

Yellow crystals (78% yield); mp > 220 °C; ^1^H NMR (400 MHz, DMSO-*d*_6_) δ 6.71–6.76 (m, 2H), 7.43 (s, 1H), 7.69 (d, *J* = 8.3 Hz, 1H), 7.7–7.77 (m, 1H), 7.79–7.85 (m, 1H), 7.87 (d, *J* = 5.6 Hz, 1H), 8.03 (d, *J* = 8.1 Hz, 1H), 8.35 (d, *J* = 8.9 Hz, 1H), 8.69 (d, *J* = 5.6 Hz, 1H), 11.3 ppm (s, 1H); ^13^C NMR (100 MHz, DMSO-d_6_) δ 98.78, 105.12, 112.39, 113.23, 120.99, 125, 126.42, 127.44, 128.25, 130.59, 135.8, 142.65, 149.81, 151.34, 167.15, 169.05, 182.01 ppm; MS (ACPI) *m/z 2*90.2 (MH^+^, 100); HRMS (ESI/HESI) *m/z:* [M + H]^+^ Calcd for C_18_H_11_NO_3_ 290.0812; Found 290.0810.

### (2*Z*)-6-Hydroxy-2-(quinolin-2-ylmethylene)-1-benzofuran-3(2*H*)-one (4b)

Yellow crystals (72% yield); mp 249–251 °C; ^1^H NMR (400 MHz, DMSO-d_6_) δ 6.75 (dd, *J* = 8.4, 2 Hz, 1H), 6.78–6.9 (m, 2H), 7.56–7.73 (m, 2H), 7.75–7.88 (m, 1H), 7.93–8.13 (m, 2H), 8.29 (d, *J* = 8.7 Hz, 1H), 8.48 (d, *J* = 8.7 Hz, 1H), 11.39 ppm (s, 1H); ^13^C NMR (126 MHz, DMSO-d_6_) δ 98.84, 110, 112.29, 113.41, 122.62, 126.29, 126.88, 127.47, 127.79, 129.08, 130.11, 136.72, 147.82, 149.55, 151.88, 167.13, 168.36, 181.53 ppm; MS (ACPI) *m/z* 290.0 (MH^+^, 100); HRMS (ESI/HESI) *m/z:* [M + H]^+^ Calcd for C_18_H_11_NO_3_ 290.0812; Found 290.0806.

### (2*Z*)-6-Hydroxy-2-[(8-methoxyquinolin-2-yl)methylene]-1-benzofuran-3(2*H*)-one (4c)

Yellow crystals (68% yield); mp 250–252 °C; ^1^H NMR (400 MHz, DMSO-d_6_) δ 4 (s, 3H), 6.75 (dd, *J* = 8.5, 2 Hz, 1H), 6.81 (s, 1H), 6.85 (d, *J* = 2 Hz, 1H), 7.09–7.30 (m, 1H), 7.45–7.62 (m, 2H), 7.67 (d, *J* = 8.5 Hz, 1H), 8.3 (d, *J* = 8.7 Hz, 1H), 8.42 (d, *J* = 8.7 Hz, 1H), 11.38 ppm (s, 1H); ^13^C NMR (100 MHz, DMSO-d_6_) δ 55.75, 98.59, 109.12, 110.09, 112.2, 113.21, 119.05, 122.67, 125.9, 127.71, 127.77, 136.19, 139.81, 149.11, 150.13, 155.13, 166.87, 168.12, 181.22 ppm; MS (ACPI) *m/z* 320.0 (MH^+^, 100); HRMS (ESI/HESI) *m/z:* [M + H]^+^ Calcd for C_19_H_13_NO_4_ 320.0917; Found 320.0919.

### (2*Z*)-2-[(1-Ethyl-5-methoxy-1*H*-indol-3-yl)methylene]-6-hydroxy-1-benzofuran-3(2*H*)-one (4d)

Yellow crystals (77% yield); mp 265–267 °C; ^1^H NMR (400 MHz, DMSO-*d*_6_); δ 1.39 (t, *J* = 7.2 Hz, 3H), 3.85 (s, 3H), 4.27 (q, *J* = 7.2 Hz, 2H), 6.72 (dd, *J* = 8.4, 2 Hz, 1H), 6.83 (d, *J* = 2 Hz, 1H), 6.87 (dd, *J* = 8.9, 2.4 Hz, 1H), 7.23 (s, 1H), 7.42 (d, *J* = 8.8 Hz, 1H), 7.56–7.64 (m, 2H), 8.18 (s, 1H), 10.98 ppm (s, 1H); ^13^C NMR (126 MHz, DMSO-*d*_6_); δ 15.38, 41.3, 55.46, 98.49, 101.1, 105.37, 107.42, 111.41, 112.56, 112.76, 114.38, 125.36, 128.22, 130.74, 133.5, 144.76, 155.02, 165.47, 166.52, 180.12 ppm; MS (ACPI) *m/z* 336.0 (MH^+^, 100); HRMS (ESI/HESI) *m/z:* [M + H]^+^ Calcd for C_20_H_17_NO_4_ 336.1230; Found 336.1224.

### (2*Z*)-6-Hydroxy-2-(4-pyrrolidin-1-ylbenzylidene)-1-benzofuran-3(2*H*)-one (4e)

Yellow crystals (83% yield); mp > 220 °C; ^1^H NMR (400 MHz, DMSO-*d*_6_) δ 1.83–2.07 (m, 4H), 3.26–3.32 (m, 4H), 6.61 (d, *J* = 8.9 Hz, 2H), 6.66–6.72 (m, 2H), 6.77 (d, *J* = 1.9 Hz, 1H), 7.57 (d, *J* = 8.4 Hz, 1H), 7.77 (d, *J* = 8.9 Hz, 2H), 11 ppm (s, 1H); ^13^C NMR (100 MHz, DMSO-*d*_6_) δ 24.95, 47.24, 98.42, 111.97, 112.58, 112.94, 113.69, 118.62, 125.39, 133.16, 144.73, 148.49, 165.58, 166.87, 180.64 ppm; MS (ACPI) *m/z* 308.1 (MH^+^, 100); HRMS (ESI/HESI) *m/z:* [M + H]^+^ Calcd for C_19_H_17_NO_3_ 308.1281; Found 308.1279.

### (*Z*)-2-((2-((1-Ethyl-5-methoxy-1*H*-indol-3-yl)methylene)-3-oxo-2,3-dihydrobenzofuran-6-yl)oxy)acetonitrile (5a)

To a solution of 670 mg (2 mmol) of (2*Z*)-2-[(1-ethyl-5-methoxy-1*H*-indol-3-yl)methylene]-6-hydroxy-1-benzofuran-3(2*H*)-one (**4d**) in 10 mL of DMF was added 830 mg (6 mmol, 3 eq) of anhydrous potassium carbonate. The mixture was heated to 60 °C and 0.152 mL (2.4 mmol, 1.2 eq) of chloroacetonitrile was added. The mixture was stirred at 60 °C for an additional 8 h, cooled, and poured into 100 mL of 0.1 N aqueous sulfuric acid. The precipitate was collected by filtration, washed with water, dried and re-crystallized from DMF-methanol to afford 487 mg (65%) of **5a** as yellow crystals: mp 230–232 °C; ^1^H NMR (400 MHz, DMSO-d_6_) δ 1.44 (d, *J* = 7.2 Hz, 3H), 3.86 (s, 3H), 4.33 (q, *J* = 7.2 Hz, 2H), 5.39 (s, 2H), 6.9 (dd, *J* = 8.9, 2.4 Hz, 1H), 6.97 (dd, *J* = 8.6, 2.2 Hz, 1H), 7.29 (d, *J* = 2.2 Hz, 1H), 7.37 (s, 1H), 7.51 (d, *J* = 8.9 Hz, 1H), 7.63 (d, *J* = 2.4 Hz, 1H), 7.77 (d, *J* = 8.6 Hz, 1H), 8.23 ppm (s, 1H); ^13^C NMR (100 MHz, DMSO-d_6_) δ 14.67, 40.97, 53.89, 55.34, 98.17, 101.45, 106.18, 107.17, 110.99, 111.68, 112.46, 115.45, 116.87, 124.84, 127.89, 130.8, 133.46, 144.2, 154.96, 162.6, 165.49, 179.58 ppm; MS (ACPI) *m/z* 375.2 (MH^+^, 100); HRMS (ESI/HESI) *m/z:* [M + H]^+^ Calcd for C_22_H_19_N_2_O_4_ 375.1339; Found 375.1337.

### (2*Z*)-6-[(2,6-Dichlorobenzyl)oxy]-2-(pyridin-4-ylmethylene)-1-benzofuran-3(2*H*)-one (5b)

To a solution of 1.5 g (10 mmol) of 6-hydroxybenzofuran-3(2*H*)-one (**2)** in 30 mL of DMF was added 4.14 g (30 mmol, 3 eq) of anhydrous potassium carbonate followed by 2.35 g (12 mmol, 1.2 eq) of 2,6-dichlorobenzyl chloride (Thermofisher Acros Organics, Geel, Belgium). The mixture was stirred at 25 °C for 8 h and diluted with 200 mL of water. The precipitate was collected, washed with water, dried and purified by column chromatography using 1:100 dichloromethane-methanol to afford 1.79 g (58%) of 6-((2,6-dichlorobenzyl)oxy)benzofuran-3(2*H*)-one as pale yellow crystals: mp 153–155 °C. ^1^H NMR (400 MHz, CDCl_3_) δ 4.64 (s, 2H), 5.34 (s, 2H), 6.67–6.77 (m, 2H), 7.29 (d, *J* = 7.2 Hz, 1H), 7.33–7.42 (m, 2H), 7.58 (d, *J* = 9 Hz, 1H); ^13^C NMR (100 MHz, CDCl_3_) δ 65.57, 75.56, 97.32, 111.98, 114.76, 125.15, 128.56, 130.9, 130.96, 136.97, 167.18, 176.32, 197.49 ppm; MS (ACPI) *m/z* 309.2 (MH^+^, 100). To 50 mL of a freshly prepared 0.2 M (5 eq) solution of sodium methoxide was added a solution of 618 mg (2 mmol) of 6-((2,6-dichlorobenzyl)oxy)benzofuran-3(2*H*)-one and 214 mg (2 mmol, 1 eq) of 4-pyridinecarboxaldehyde in 5 mL of methanol. The mixture was stirred at 25 °C for 12 h. The solution was concentrated and poured into 100 mL of water at 0 °C. The mixture was acidified with 1N aqueous hydrochloric acid solution to *ca*. pH 6. The precipitate was collected by filtration and recrystallized from 2:1 DMF-methanol to afford 445 mg (56%) of **5b**: mp 219–222 °C; ^1^H NMR (400 MHz, CDCl_3_) δ 5.41 (s, 2H), 6.7 (s, 1H), 6.88 (dd, *J* = 8.6, 2.2 Hz, 1H), 6.96 (d, *J* = 2.2 Hz, 1H), 7.28–7.36 (m, 1H), 7.36–7.45 (m, 2H), 7.68–7.78 (m, 3H), 8.7 ppm (d, *J* = 5.2 Hz, 2H); ^13^C NMR (126 MHz, CDCl_3_) δ 66.04, 98, 108.3, 113.23, 114.8, 124.74, 126.45, 128.78, 130.92, 131.17, 137.19, 139.95, 150.3, 150.36, 167.19, 168.85, 182.73 ppm; MS (ACPI) *m/z* 398.0 (MH^+^, 100); HRMS (ESI/HESI) *m/z:* [M + H]^+^ Calcd for C_21_H_13_Cl_2_NO_3_ 398.0345; Found 398.0349.

### Biological Studies

PC-3, MCF-7 and A549 cells were cultured in the medium recommended by American Type Culture Collection at 37 °C with 5% CO_2_ atmosphere in a water jacketed incubator (NuAire). Ovcar-8 and NCI/ADR-RES cells were gifts from Dr. Markos Leggas, University of Kentucky, Lexington, KY USA. The beta-tubulin antibody was from Developmental Studies Hybridoma Bank. (Iowa city, IA USA).

### Cell proliferation inhibition assay

Cancer cells were seeded into 24-well plates at a density of 20,000 cells per well in 1 mL of culture medium and were cultured overnight at 37 °C. Compounds and the vehicle control (DMSO) were added to the cells. After 6 days, the medium was removed, and 100 µL of trypsin was added. The cells were re-suspended in phosphate-buffered saline (PBS) and were counted by Vi-CELL XR 2.03 (Beckman Coulter, Inc. USA). The ratio R of the number of viable cells in the compound treatment group to the number of viable cells in DMSO treatment group was taken as relative growth, and the percentage growth inhibition was calculated as (1 − R)*100. For initial testing, compounds were added to the cells at a final concentration of 10 µM. Active compounds at 10 µM were tested at lower concentrations than 10 µM.

### *In vitro* tubulin polymerization assay

An *in vitro* tubulin polymerization assay was performed using a protocol from Cytoskeleton, Inc. (Denver, CO USA). Tubulin powder (Cytoskeleton Inc. Denver, CO USA) was dissolved in a buffer prepared from 100 mM PIPES (pH 6.9), 2 mM MgCl_2_, 1 mM GTP, and 5% glycerol at 0 °C. Aliquots (80 µL, 3.75 µg/µL) of this tubulin solution were divided into the wells of a 96-well half-area plate (Corning Inc., NY USA). After adding either DMSO or testing compounds, the plate was mounted on a Spectra MR^TM^ microplate spectrophotometer equipped with a thermal controller at 37 °C (Dynex Technologies, Inc., Chantilly, VA USA). Readings at 350 nm were recorded every 30 s for 1 h.

### *In vivo* microtubule assembly assay

The amount of insoluble polymerized microtubules and soluble tubulin dimers in cells after exposure to aurones were detected using a reported method. Cells were seeded in 6-well plates at 50% confluency and cultured overnight. DMSO or aurones in DMSO solution were added, and the cells were incubated for additional 6 h. The medium was removed, and cells were washed with PBS three times followed by the addition of a lysis buffer prepared from 20 mM Tris-HCl (pH 6.8), 1 mM MgCl_2_, 2 mM EGTA, 20 µg/mL aprotinin, 20 µg/mL leupeptin, 1 mM PMSF, 1 mM orthovanadate, and 0.5% NP40. The lysates were centrifuged at 12,000 g for 10 min to obtain supernatants and pellets that were mixed with loading buffer and heated to 100 °C. Standard western blotting against α-tubulin was performed as described previously^56^.

### Immunofluorescence imaging

Tubulin networks were examined by confocal immunofluorescence imaging. Briefly, PC3 cells were placed at a density of 80,000/mL to 24-well plates equipped with round microscope glass cover slides. After culturing at 37 °C for 24 hours, DMSO or compounds were added to the cells and incubated for additional 6 hours. Then the medium was removed and the cells were washed with PBS three times. Primary anti-α-tubulin antibody was added and incubated overnight at 4 °C. After additional washing, secondary TRITC-conjugated anti-rabbit antibody was added for 40 min, followed by additional washing and staining with DAPI. Final washing was performed and the cover slides were inverted onto glass slides. Images (40x) were taken using a Nikon confocal microscope with excitation at 557 nm and emission at 576 nm.

### Molecular docking studies

An X-ray crystal structure of αβ-tubulin binding with colchicine (pdb: 4O2B) was downloaded from RCSB Protein Data Bank and manipulated using AutoDockTools-1.5.6 (Molecular Graphics Laboratory, The Scripps Research Institute, La Jolla, CA 92037 USA).The αβ-Tubulin dimer was separated from 4O2B using PyMOL (Version 1.7.4.5 Edu). Water molecules were removed, and polar hydrogens and Kollman charges were added. The docking pocket (colchicine-binding site) was defined as follows: Search space: 18 × 18 × 18 Å^3^; Center_x, y, z = 14.815, 9.422, −20.186. The aurones **4d**, **5a**, and colchicine were manipulated by Openbabel. Molecular docking of **4d**, **5a**, and colchicine to the colchicine-binding site was executed using AutoDock vina-1.1.2 using the iterated gradient-based local search method with a Broyden–Fletcher–Goldfarb–Shanno (BFGS) method for local optimization^43^. Exhaustiveness was set at 14 and the number of modes was nine. Other parameters were left at default values.

### hERG binding studies

An HEK-293 cell line stably expressing the hERG potassium channel (accession number U04270) referred to as hERG-HEK cells were received at passage 11 (P11) from Millipore (CYL3006, lot 2, Billerica, MA USA). [^3^H]-Dofetilide (specific activity of 80 Ci/mmol; labeled on the N-methyl group) was obtained from American Radiolabeled Chemicals, St. Louis, MO USA). Other chemicals and solvents were obtained from Sigma-Aldrich (Milwaukee, WI USA) with exceptions of polyethylenimine (PEI), which was obtained from Fluka/Sigma-Aldrich (St. Louis, MO USA), and Minimium Essential Medium (MEM) with GlutaMAX^TM^ and phenol red, MEM non-essential amino acids solution (NEAA, 100X), G418 disulfate salt solution, fetal bovine serum (FBS), 0.05% Trypsin-EDTA 1X with phenol red, and Hank’s balanced salt solution (HBSS), which were obtained from Life Technologies (Carlsbad, CA USA).

### hERG-HEK Cell Culture

The hERG-HEK cells were cultured according to the protocol provided by Merck Millipore (Burlington, MA USA). Cells were maintained in MEM (with glutamax and phenol red) supplemented with 10% FBS, 1% NEAA and 400 μg/ml geneticin, and incubated at 37 °C in a humidified atmosphere with 5% CO_2_. Frozen aliquots of cells were transferred into T-75 cm^2^ flasks and allowed to adhere for 4–8 h. The medium was replaced every 2 days. Passages were carried out at least 3 times after thawing at 6 day intervals. Cells were dissociated with trypsin/EDTA and seeded into new 150 × 25 mm dishes at 2–3 × 10^6^ cells per dish and placed at 30 °C, 5% CO_2_, for 40–48 h prior to membrane preparation. Membrane preparation occurred 6 days after the last passage (passage 20).

### Membrane preparation

Cell membrane preparation was based on previous methods^49,50,57^. Cells were rinsed twice with HBSS at 37 °C and collected by scraping the dishes in *ca*. 20 mL of ice-cold 0.32 M sucrose and homogenized on ice with a Teflon pestle using a Maximal Digital homogenizer (Fisher Scientific, Pittsburgh, PA USA) at ~280 rpm for 30 sec. Homogenates were centrifuged at 300 g and 800 g for 4 min each at 4 °C. Pellets were resuspended in 9 mL of ice-cold Milli-Q water and osmolarity restored by addition of 1 mL of 500 mM Tris buffer (pH 7.4) followed by suspension and centrifugation at 20,000 g for 30 min at 4 °C. Pellets were homogenized in 2 mL assay buffer (50 mM Tris, 10 mM KCl, and 1 mM MgCl_2_, 4 °C) and aliquots of cell membrane suspensions were stored at −80 °C and thawed the day of the [^3^H]-dofetilide binding assay. Protein content was determined prior to the assay using a Bradford protein assay with bovine albumin as the standard.

### [^3^H]-Dofetilide binding assay

[^3^H]-Dofetilide binding assays using hERG-HEK293 cell membranes were based on previous methods. Assays determining concentration-response were performed in duplicate, and three independent assays were performed for each analog evaluated. Cell membrane suspension (5 μg) was added to duplicate tubes containing assay buffer, 25 μL of a single concentration of FIDAS agent (concentration range of 10 nM-100 µM for each experiment), and 25 μL of [^3^H]-dofetilide (5 nM, final concentration) for an assay volume of 250 μL. Binding occurred for 60 min at 25 °C and was terminated by rapid filtration through Whatman GF/B filters, which were pre-soaked in 0.25% PEI overnight, using a Brandel cell/membrane harvester (M-48; Brandel Inc., Gaithersburg, MD USA). Filters were washed three times with *ca*. 1 mL of ice-cold assay buffer. Radioactivity was determined by liquid scintillation spectrometry using the Tri-Carb 2100-TR Liquid Scintillation Analyzer (Perkin-Elmer Life and Analytical Sciences).

### *In vivo* evaluation of anti-leukemia activity in the zebrafish model

Zebrafish studies were carried out with approval from the Institutional Animal Care and Use Committees of the University of Kentucky (2015–2225). All methods were performed in accordance with the relevant guidelines and regulations according to protocols. *Rag2*: *myc-GFP* zebrafish (n = 8) at 21 days of age were treated with DMSO, either aurones **5a** or **5b** in 1.5 mL of fish-system water in 12-well plates. Zebrafish were treated with compound for 2 days, removed from drug for 1 day, and treated for two more days with freshly prepared solutions of compound. Animals were imaged at the start and end of treatment using a fluorescence-equipped dissecting microscope at 350 ms exposure. The GFP image was overlaid onto the bright-field image of each animal in Photoshop, and the percent change in leukemia burden was calculated by normalizing the GFP+ area to the total area of the animal in ImageJ (National Institute of Health, USA).

### *In vivo* evaluation of anti-cancer activity and gross toxicity in PC-3 xenografts

Mouse studies were carried out with approval from the Institutional Animal Care and Use Committees of the University of Kentucky (2009–1064). All methods were performed in accordance with the relevant guidelines and regulations according to protocols. PC-3 cells suspended in PBS were subcutaneously injected in the lower flanks of immune-deficient nude mice (5 mice in each group, two tumors on each mouse) at a density of 2 × 10^6^ cells in 200 μL of PBS. After tumors were established (in about two weeks), aurone **5a** formulated in a mixture of Tween-80 (5%), DMSO (10%), PEG400 (25%) and PBS (60%) was intraperitoneally administered to mice at a daily dose of 10 mg of aurone **5a**/kg (mouse). The first day of treatment was set as day 1. At day 18 treatment was ceased and mice were sacrificed. Blank vehicle was used as a control. Tumors and mouse weights were measured, and tumor volumes were calculated as Length × width^2^/2. For H&E and IHC studies, the tumors were treated with vehicle and aurone **5a** for 1 week. H&E and TUNEL staining was performed based on standard protocol by the Markey Cancer Center Biospecimen Procurement & Translational Pathology Shared Resource Facility (BPTP SRF) at the University of Kentucky. For IHC staining, the following antibody was used: anti- VEGF-A (Santa Cruz, sc-152, 1:100).

### Competitive tubulin binding assay and Liquid Chromatography-Electrospray Ionization-Tandem Mass Spectrometry (LC-ESI-MS/MS) method

Competitive tubulin binding assay was performed as described to demonstrate that aurones bind to the colchicine-binding site of tubulins. The colchicine quantification was performed at the University of Kentucky Proteomics Core using a protocol modified from a previously published method. LC-MS/MS analysis was carried out using an TSQ Vantage mass spectrometer (Thermo Fisher Scientific, Waltham, MA USA) coupled with a Shimadzu high performance liquid chromatography (HPLC) system (Shimadzu Scientific Instruments, Inc., Columbia, MD USA) through an electrospray ionization source. The colchicine-containing samples were separated with a Kinetex^*®*^ reversed phase 2.6 μm XB-C18 100 Å LC column (100 × 4.6 mm) (Phenomenex Inc., Torrance, CA USA) at a flow rate of 300 μL/min. Mobile phase A was water with 0.1% (*v*/*v*) formic acid while mobile phase B was acetonitrile with 0.1% (*v*/*v*) formic acid. A 16 min gradient condition was applied: initial 60% mobile phase B was increased linearly to 100% in 3 min, remained 100% for 3 min, and quickly (0.01 min) decreased to 60% for re-equilibration. Multiple reaction monitoring (MRM) mode was used to scan from m/z 400 to m/z 310 in the positive mode to obtain the most sensitive signals for colchicine. The spraying voltage was set at 4000 V, vaporize temperature at 300 °C, capillary temperature at 350 °C and sheath gas pressure at 45 (arbitrary units). Collision energies (CE) were set at 25 volts. Xcalibur software (Ver. 2.1.0, Thermo Fisher Scientific, Waltham, MA USA) was used for the data acquisition and quantitative processing. A series of colchicine at concentrations of 20, 40, 80, 200, 400, 800 nM were prepared to establish a linear calibration curve with a coefficient of correlation R^2^ = 0.9944.

### Statistics

Biological assays have been performed at least twice. Data were shown as mean ± SD or the 95% confidence intervals were provided. For the mice study, five mice with two tumors on the lower flanks of each mouse were used in each treatment group. The data for the mouse study were analyzed by t-test. For the zebrafish study, eight fish were used in each treatment group.

## Supplementary information

Supplementary info

## Data Availability

The data related to this manuscript during the current study are available from the corresponding authors on reasonable request.
