# Supplementary material for: Semisynthetic aurones inhibit tubulin polymerization at the colchicine-binding site and repress PC-3 tumor xenografts in nude mice and myc-induced T-ALL in zebrafish
Source: Sci Rep. 2019 Apr 23;9:6439. doi: 10.1038/s41598-019-42917-0 (PMC6478746; doi:10.1038/s41598-019-42917-0)
Supplement: Supplementary file 1 — Supplementary info [file 41598_2019_42917_MOESM1_ESM.pdf]

Semisynthetic aurones inhibit tubulin polymerization at the colchicine-binding site and repress PC-3 tumor xenografts in nude mice and myc-induced T-ALL in zebrafish

Yanqi Xie[1,2], Liliia M. Kril[1,2], Tianxin Yu[1,3], Wen Zhang[1,3], Mykhaylo S. Frasinyuk[1,2,4], Svitlana P. Bondarenko[5], Kostyantyn M. Kondratyuk[4], Elizabeth Hausman[1], Zachary M. Martin[1,2], Przemyslaw P. Wyrebek[1,2], Xifu Liu[6], Agripina Deaciuc[7], Linda P. Dwoskin[7], Jing Chen[1], Haining Zhu[1], Chang-Guo Zhan[2,7,8], Vitaliy M. Sviripa[2,3,7], Jessica Blackburn[1], David S. Watt[1,2,3,7*] & Chunming Liu[1,3*]

[1]Department of Molecular and Cellular Biochemistry, College of Medicine, University of Kentucky, Lexington, KY 40536-0509, USA. [2]Center for Pharmaceutical Research and Innovation, College of Pharmacy, University of Kentucky, Lexington, KY 40536-0596, USA [3]Lucille Parker Markey Cancer Center, University of Kentucky, Lexington, KY 40536-0093, USA [4]Institute of Bioorganic Chemistry and Petrochemistry, National Academy of Science of Ukraine, Kyiv 02094, Ukraine [5]National University of Food Technologies, Kyiv, 01601, Ukraine [6]Center for Drug Innovation and Discovery, Hebei Normal University, Shijiazhuang, Hebei 050024, People's Republic of China [7]Department of Pharmaceutical Sciences, College of Pharmacy, University of Kentucky, Lexington, KY 40536-0596, USA [8]Molecular Modeling and Pharmaceutical Center, College of Pharmacy, University of Kentucky, Lexington, KY 40536-0596, USA. Correspondence and requests for materials should be addressed to CL (email: dwatt@uky.edu; chunming.liu@uky.edu)

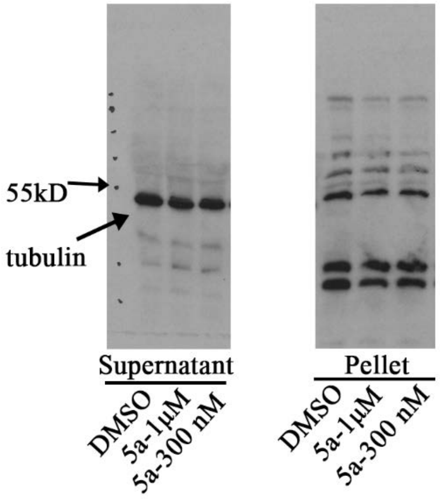

Supplementary Figure 1, uncut gel for Figure 3C in manuscript.
